# Association between lifestyle and COVID-19 vaccination: A national cross-sectional study

**DOI:** 10.3389/fpubh.2022.918743

**Published:** 2022-10-11

**Authors:** Yudong Miao, Wanliang Zhang, Yi Li, Jian Wu, Dongyang Xu, Jianqin Gu, Meiyun Wang, Wei Wei, Beizhu Ye, Chengyuan Miao, Clifford Silver Tarimo, Wenyong Dong

**Affiliations:** ^1^Department of Health Management, College of Public Health, Zhengzhou University, Zhengzhou, China; ^2^Henan Research Center for He'nan Institute for Health Economy and Health Technology Assessment (HTA), Zhengzhou, China; ^3^Research Center for Lifestyle Medicine, School of Medicine, Southern University of Science and Technology, Shenzhen, China; ^4^Henan Provincial People's Hospital, People's Hospital of Zhengzhou University, Zhengzhou, China

**Keywords:** COVID-19, COVID-19 vaccination, lifestyle, propensity score matching, China

## Abstract

**Objective:**

To assess lifestyles, COVID-19 vaccination coverage rates, and the relationships between lifestyles and COVID-19 vaccination among Chinese population.

**Methods:**

We collected data on sociodemographics, perception of the COVID-19 pandemic, lifestyles, and self-reported COVID-19 vaccination *via* an online survey in China. The chi-square goodness-of-fit test was used to monitor sample saturation throughout the formal online survey. The binary logistic regression analyses were conducted to examine the association between COVID-19 vaccination rate and lifestyle score. We assigned values to 12 lifestyles ranging from positive to negative, with positive lifestyles receiving a higher score and negative lifestyles receiving a lower score, ranging from 1 to 5. For each participant, the total lifestyle scored from 12 to 56. Restricted cubic spline (RCS) was used to visualize the trends and correlations between lifestyle score and COVID-19 vaccination coverage. Propensity score matching (PSM) was used to explore the association between specific lifestyles and COVID-19 vaccination.

**Results:**

A total of 29,925 participants (51.4% females) responded. The lifestyle score of the sample was 44.60 ± 6.13 (scoring range: 12–56). COVID-19 vaccination rate was found to be 89.4% (89.1–89.8%). Female participants reported a higher vaccination rate than male participants (91.5 vs. 87.1%). Compared to Q1, COVID-19 vaccination coverage rates increased with lifestyle total scores [OR_*Q*2_ = 1.901 (1.718–2.103), *P* < 0.001; OR_*Q*3_ = 2.373 (2.099–2.684), *P* < 0.001; and OR_*Q*4_ = 3.765 (3.209–4.417), *P* < 0.001]. After applying PSM, it was determined that all the 12 specific healthy lifestyles analyzed, including maintaining a healthy body weight, a healthy diet, regular physical exercises, adequate sleep, regular physical examination, and others, were found to be positive factors for COVID-19 vaccination.

**Conclusion:**

The majority of mainland Chinese lived a healthy lifestyle throughout the COVID-19 pandemic, and the rate of COVID-19 vaccination was high. Specific healthy lifestyles contributed to COVID-19 vaccination coverage rates significantly. According to the study's findings, global efforts to achieve herd immunity should be prioritized by continually promoting healthy lifestyles and improving public perception of COVID-19 vaccines.

## Introduction

Vaccination is the most cost-effective method of preventing infectious diseases and has historically been one of the most effective public health interventions (1–5). Since the global outbreak of COVID-19, researchers from all around the world have been working tirelessly and collaboratively to develop vaccines against the virus. Numerous vaccinations have been developed to prevent and control COVID-19, including messenger RNA (mRNA) vaccines, DNA vaccines, and inactivated vaccines (6). In the current stage, there is still a gap between the COVID-19 vaccination rate and the herd immunization target in most countries. Despite the introduction of various vaccinations types, the vaccination coverage rate remains low (7, 8). Studies indicate that 10% of Americans refused to get vaccinated, while 30% were hesitant to get vaccinated against COVID-19 (9). Another study conducted in Russia found 55% refusal rate (10), and 28.3% were hesitant about COVID-19 vaccine (11). The ongoing low COVID-19 vaccination coverage rate is hampering the vaccination coverage rate required to achieve herd immunity globally (12), and, hence, diminishing the social benefits of vaccinating against COVID-19 (13, 14).

The reasons for the low rate of COVID-19 vaccination coverage are numerous and, to some extent, ambiguous. Earlier research examined the complexities of vaccination coverage rates by focusing on the epidemiologic triad including environmental, agent, and host factors (15). Environmental factors include public health policies, social factors, and media messaging (16, 17), whereas agent (vaccine and disease) factors include perceived vaccine safety and effectiveness, in addition to perceived disease susceptibility (18). The host factors are contingent upon one's knowledge, prior experience, educational attainment, and income level (19). Recent research indicates that vaccination coverage rate may be framed in terms of complacency, confidence, and convenience (3Cs framework). Vaccination occurs when there is a high perception of the necessity for vaccination (referred to as complacency), trust in the efficacy and safety of the vaccine (referred to as confidence), and vaccine accessibility (referred to as convenience) (20). Based on the frameworks, youth, female gender, income, education, informational reliance on social media, informational reliance on print and broadcast media, ethnicity, perceived risk from COVID-19 and trust in scientists, medics and biomedical science, as well as trust in government were all recognized as relevant factors that may affect COVID-19 vaccination coverage. In addition, some studies had shown that specific lifestyles such as smoking, obesity, and exercise also could affect COVID-19 vaccination based on this framework: for instance, smoking and obesity may cause people fearing of side effects and weaken confidence and to be reluctant to vaccinate, while exercise improved the physical fitness of people and made people complacent and to be reluctant to vaccinate, and people with frequent physical examinations may be more familiar with the location of vaccination sites than those who do not, which also helps to increase the geographical accessibility of vaccination, which may affect vaccination (21–24). To date, though these studies mainly explored the relationship between behaviors such as smoking, drinking, and COVID-19 vaccination, there is yet little global research on assessing COVID-19 vaccination rate and exploring the association between other lifestyles such as washing hands, wearing masks, and COVID-19 vaccination.

The purpose of this study was to create a realistic scenario of how the Chinese live, their vaccination uptake, and the effect of their lifestyle on vaccination coverage. We, therefore, conducted a nationwide survey in 31 provinces throughout mainland China during the period of primary and booster COVID-19 vaccine vaccination. We estimated COVID-19 vaccination coverage in a large sample by analyzing self-reported vaccination status and identifying subgroups within the population that may have a higher rate. Our primary goal was to gain a better understanding of vaccination coverage rates from a lifestyle standpoint in order to guide future vaccination coverage rate increase.

## Methods

### Participants and procedures

We performed a preliminary online survey in Zhongmu County, Henan Province, on 10 July 10 2021. We made face-to-face interviews with participants from a representative village and community using the cluster sampling method. Based on the preliminary vaccination rate and the validity and reliability of the preliminary online questionnaire, we estimated that a formal survey required a minimum sample size of 6,638 participants (we used α of 0.05 and Zα/2 of 1.959964), the details on sample size estimation as follows:


(1) $$\begin{array}{l} n=\frac{Z_{\alpha /2}^{2}*P*\left(1-P\right)}{d^{2}} \end{array}$$

The minimum sample size based on the COVID-19 vaccination coverage rate of 83.43% in the preliminary online survey, an allowable error of 1%, and consider the missing 20% sample size.

**Figure 1:**
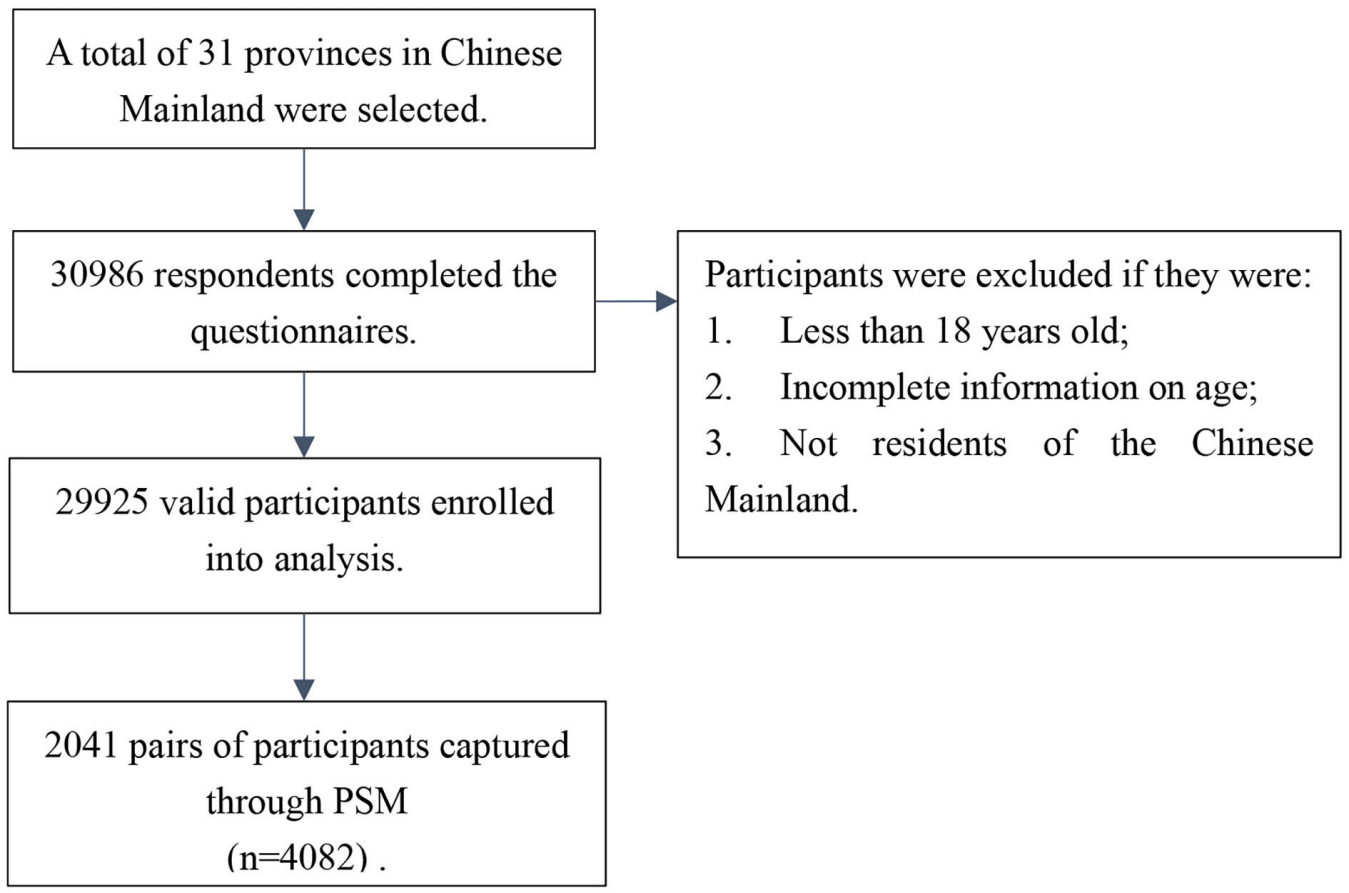
The flowchart of participant selection.

Due to the domestic epidemic of COVID-19, we cannot conduct face-to-face surveys; then, on 6 August 2021, we performed a nationwide cross-sectional online survey among Chinese adults (≥18 years) using snowball sampling *via* an online survey company. To ensure that our sample size was sufficient to reliably estimate the vaccination rate and to ensure our sample was sufficiently representative, we used and monitored sample saturation throughout the survey. In this study, sample saturation referred to the point at which the vaccination rate becomes stable and does not change appreciably as the sample size increases (see Annexure 1: Figure 1). The sample became saturated when the sample size reached 20,990. We ended the online survey when 29,925 eligible questionnaires were acquired on 9 August 2021, comprising sociodemographic variables, the perception of COVID-19, lifestyles, and self-report vaccination. A brief flowchart of participant selection is shown in Figure 1. The study protocol and online survey were approved by the Life Science Ethics Review Committee of the Zhengzhou University (record no: 2021-01-12-05).

**Figure 2:**
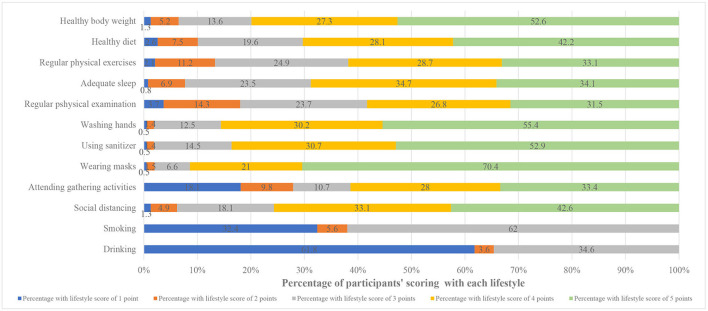
The overall composition of participants' scoring by each lifestyle.

All the online users (all the participants) have read and agreed to the informed consent (please see the informed consent in Annexure 2: Figure 2).

### Assessments

The purpose of the current study was to understand the lifestyle of Chinese mainland residents in the context of COVID-19 outbreak, COVID-19 vaccination coverage rate, and their relationships. Data were collected from four aspects (for the data collection and explanatory variables, please see details in Annexure 3: Table 1).

(1) Sociodemographic characteristics (including gender, age, occupation status, marital status, and education status).

(2) The perception of COVID-19 [including the negative predictive value (NPV) mutation, the effectiveness of COVID-19 vaccine, and the protection period of COVID-19 vaccine].

(3) Lifestyles: We reviewed previous articles about lifestyle and then developed a questionnaire to assess lifestyle based on the American Medical Association Healthy Lifestyle scale and the Likert 5-point scale. The lifestyle section of the questionnaire contained 12 lifestyle behaviors, including healthy body weight, healthy diet, regular physical exercises, adequate sleep, regular physical examination, handwashing, using sanitizers, wearing masks, gathering activities, social distancing, smoking, and drinking (for the specific questions and classification details of the 12 lifestyle behaviors in the questionnaire, please refer to Annexure 3: Table 1). In terms of lifestyle score, lifestyle 1–8 and 10 scored 1 to 5 (never = 1, little = 2, sometimes = 3, often = 4, and always = 5; significantly decrease = 1, decrease = 2, no change = 3, increase = 4, and significantly increase = 5); lifestyle 9 scored 1–5 (significantly decrease = 5, decrease = 4, no change = 3, increase = 2, and significantly increase = 1); and lifestyle 11–12 scored 1 to 3 (regular = 1, quit = 2, and never = 3). For each participant, their total lifestyle scored 12–56.

(4) Self-reported COVID-19 vaccination: The questionnaire included a question about whether the respondent had received the primary COVID-19 vaccine; this question was designed to elicit information about vaccination rates. For this item, item-specific response options coded ranging from 1–5 were used, including: (a) vaccinated, (b) being vaccinated, (c) no, but preparing to receive the COVID-19 vaccine, (d) no, and not sure to get the COVID-19 vaccine, and (e) no, and hesitant to receive the COVID-19 vaccine. During the analysis, options (a) and (b) were merged into “vaccinated” whereas options (c), (d), and (e) were merged into “unvaccinated.”

### Statistical analysis

The chi-square goodness-of-fit test was used to monitor sample saturation throughout the formal online survey to determine sample underrepresentation error. We used COVID-19 vaccination rates to represent behaviors that have been vaccinated against COVID-19. An independent samples *t*-test or chi-squared test was carried out to test statistical differences in COVID-19 vaccination rate and lifestyle score across groups. The binary logistic regression analyses were conducted to examine the association between COVID-19 vaccination and lifestyle score after controlling for and sociodemographical confounders. A collinearity test using the variance inflation factor (VIF) (< 4) was used to determine the correlation between independent variables. No collinearity was detected between the eight covariates. Restricted cubic spline (RCS) was used to visualize the trends and correlations between lifestyle score and COVID-19 vaccination using the 25th quantile (40 points) as the node. Further, propensity score matching (PSM) was used to explore the association between 12 specific lifestyles and COVID-19 vaccination to verify the reliability of the finds. All the statistical analyses were done using SAS version 9.4. Differences were regarded as statistically significant if *P* < 0.05.

## Results

### Lifestyle score and COVID-19 vaccination coverage rate of mainland Chinese

A total of 29,925 participants (48.6% males and 51.4% females) were responded to the questions. The overall mean score for lifestyles was 44.60 ± 6.13, whereas the mean score for males was 43.43 ± 6.24, which were lower than the mean score for the general population as well as that for females participants (45.71 ± 5.81). The overall COVID-19 vaccination coverage rate was 89.4% (89.1–89.8%). The rates were found to be 87.1% (86.6–87.7%) for males and 91.5% (91.1–92.0%) for females. Among individuals with college education or above background, the COVID-19 vaccination coverage rate was 92.2% (91.8–92.6%) with the mean lifestyle score of 45.03 ± 5.80. Students had the highest COVID-19 vaccination coverage rate (92.2%, 91.5–93.0%), but their lifestyle scores were lower on average with 43.10 ± 5.69. Regarding the perception of COVID-19, the vaccination coverage rate of individuals who were aware of the COVID-19 virus mutation and that the COVID-19 vaccine was extremely effective were 91.8% (91.5–92.1%) and 95.1% (94.7–95.4%), respectively. The mean scores of lifestyles of the groups were 45.07 ± 5.79 and 47.02 ± 5.55, respectively (see Table 1 for details). Furthermore, Figure 2 illustrates the proportion of different scores of each item, and it was apparent that the proportion of lifestyle score of 5 points was the largest.

**Table 1:** Sociodemographic characteristics, COVID-19 vaccination rate, and the lifestyle score of all the study participants.

**Covariates**	**Total participants (percentage, %)**	**Vaccination rate (%, 95% CI)**	**Lifestyle score (mean ±SD)**	**Lifestyle score (Quartile, mean** ±**SD)**				***P*-Value**
				**Q1**	**Q2**	**Q3**	**Q4**	
Total	29,925 (100)	89.4 (89.1–89.8)	44.60 ± 6.13	36.52 ± 3.17	43.12 ± 1.40	47.49 ± 1.07	52.35 ± 1.93	
**Gender**								< 0.001
Male	14,556 (48.6)	87.1 (86.6–87.7)	43.43 ± 6.24	36.24 ± 3.26	43.06 ± 1.41	47.45 ± 1.06	52.17 ± 1.85	
Female	15,369 (51.4)	91.5 (91.1–92.0)	45.71 ± 5.81	36.97 ± 2.95	43.17 ± 1.39	47.53 ± 1.07	52.46 ± 1.97	
**Age**								< 0.001
18–29	13,312 (44.5)	87.8 (87.3–88.4)	43.32 ± 6.15	36.41 ± 3.18	43.05 ± 1.40	47.46 ± 1.05	52.31 ± 1.94	
30–39	11,911 (39.8)	90.1 (89.6–90.7)	45.61 ± 5.96	36.72 ± 3.06	43.17 ± 1.40	47.52 ± 1.07	52.40 ± 1.95	
40–49	3,269 (10.9)	93.1 (92.2–94.0)	45.91 ± 5.73	36.83 ± 3.32	43.21 ± 1.40	47.51 ± 1.12	52.38 ± 1.87	
50–59	1,149 (3.8)	90.2 (88.4–91.9)	45.40 ± 5.82	36.20 ± 3.26	43.23 ± 1.35	47.45 ± 1.07	52.08 ± 1.79	
≥60	284 (0.9)	86.6 (82.6–90.6)	44.19 ± 6.53	35.06 ± 4.06	43.04 ± 1.33	47.40 ± 1.07	52.03 ± 1.76	
**Marital status**								< 0.001
Unmarried	10,533 (35.2)	89.8 (89.2–90.4)	43.30 ± 5.93	36.61 ± 3.12	43.04 ± 1.39	47.43 ± 1.04	52.24 ± 1.94	
Married	18,363 (61.4)	90.8 (90.3–91.2)	45.69 ± 5.87	36.79 ± 2.97	43.18 ± 1.40	47.52 ± 1.08	52.39 ± 1.93	
Divorced	809 (2.7)	61.7 (58.3–65.0)	38.75 ± 6.29	34.94 ± 3.33	42.80 ± 1.37	47.43 ± 1.16	52.11 ± 1.75	
Widowed	178 (0.6)	55.6 (48.2–63.0)	37.47 ± 7.37	33.33 ± 4.88	42.57 ± 1.52	47.19 ± 1.08	52.00 ± 2.06	
Others	42 (0.1)	73.8 (59.9–87.7)	39.76 ± 7.96	33.38 ± 5.27	42.60 ± 1.17	47.33 ± 1.21	51.80 ± 2.05	
**Educational level**								< 0.001
Illiteracy	257 (0.9)	65.0 (59.1–70.9)	39.51 ± 7.85	33.32 ± 5.41	43.04 ± 1.40	47.68 ± 0.87	52.73 ± 2.69	
Primary school	891 (3.0)	66.7 (63.6–69.8)	40.19 ± 6.00	35.77 ± 3.04	42.95 ± 1.41	47.44 ± 0.90	52.58 ± 2.17	
Middle school	2,691 (9.0)	81.3 (79.8–82.7)	42.72 ± 6.83	35.67 ± 3.39	43.02 ± 1.40	47.49 ± 1.05	52.33 ± 1.92	
High school	7,893 (26.4)	89.1 (88.4–89.8)	44.92 ± 6.19	36.45 ± 3.17	43.13 ± 1.42	47.54 ± 1.07	52.28 ± 1.92	
College or above	18,193 (60.8)	92.2 (91.8–92.6)	45.03 ± 5.80	36.97 ± 2.89	43.13 ± 1.39	47.47 ± 1.08	52.38 ± 1.93	
**Occupation**								< 0.001
Worker	2,951 (9.9)	91.5 (90.5–92.5)	44.90 ± 5.95	36.52 ± 3.29	43.20 ± 1.39	47.52 ± 1.06	52.15 ± 1.83	
Farmer	4,017 (13.4)	83.1 (81.9–84.2)	43.82 ± 6.69	35.98 ± 3.23	43.06 ± 1.45	47.48 ± 1.01	52.40 ± 1.90	
Business staff	6,516 (21.8)	88.1 (87.3–88.9)	45.01 ± 6.39	36.31 ± 3.22	43.15 ± 1.41	47.54 ± 1.06	52.50 ± 2.00	
Student	4,410 (14.7)	92.2 (91.5–93.0)	43.10 ± 5.69	36.87 ± 2.95	43.04 ± 1.38	47.37 ± 1.07	52.15 ± 1.94	
Technical staff	6,084 (20.3)	92.0 (91.3–92.7)	45.45 ± 5.61	37.09 ± 2.77	43.17 ± 1.38	47.49 ± 1.09	52.32 ± 1.90	
Government staff	3,387 (11.4)	91.7 (90.8–92.7)	45.69 ± 5.77	36.97 ± 2.89	43.17 ± 1.39	47.52 ± 1.08	52.48 ± 1.99	
Retired	474 (1.6)	84.0 (80.7–87.3)	44.43 ± 6.55	35.50 ± 4.22	43.02 ± 1.38	47.47 ± 1.14	52.07 ± 1.85	
No fixed occupation	1,466 (4.9)	87.2 (85.5–89.0)	43.80 ± 6.37	35.99 ± 3.60	43.06 ± 1.40	47.49 ± 1.08	52.21 ± 1.75	
Others	620 (2.0)	85.6 (82.9–88.4)	42.47 ± 6.03	36.21 ± 3.73	42.85 ± 1.43	47.42 ± 1.05	51.76 ± 1.80	
**Perception of the NPV mutation**								< 0.001
Yes	24,515 (81.9)	91.8 (91.5–92.1)	45.07 ± 5.79	36.99 ± 2.85	43.12 ± 1.40	47.50 ± 1.07	52.33 ± 1.92	
No	3,822 (12.8)	77.1 (75.7–78.4)	42.45 ± 7.25	35.28 ± 3.48	43.07 ± 1.43	47.46 ± 1.09	52.57 ± 2.00	
Unclear	1,588 (5.3)	82.2 (80.3–84.1)	42.55 ± 6.76	35.46 ± 3.88	43.09 ± 1.38	47.43 ± 1.09	52.05 ± 1.79	
**Perception of the effectiveness of COVID-19 vaccine**								< 0.001
Very effective	14,720 (49.2)	95.1 (94.7–95.4)	47.02 ± 5.55	37.06 ± 2.87	43.30 ± 1.38	47.57 ± 1.05	52.51 ± 1.96	
Effective	11,221 (37.5)	89.5 (88.9–90.1)	43.18 ± 5.36	36.89 ± 2.89	43.05 ± 1.40	47.39 ± 1.09	51.78 ± 1.70	
Not sure	2,979 (9.9)	71.9 (70.3–73.5)	40.17 ± 5.75	35.90 ± 3.40	42.79 ± 1.38	47.37 ± 1.05	51.70 ± 1.74	
Ineffective	568 (1.9)	55.1 (51.0–59.2)	37.64 ± 5.83	34.71 ± 3.64	42.90 ± 1.37	47.13 ± 0.92	51.75 ± 1.48	
Completely ineffective	200 (0.7)	46.5 (39.5–53.5)	37.63 ± 6.14	34.34 ± 3.36	42.41 ± 1.42	47.50 ± 0.92	52.50 ± 2.14	
Unclear	237 (0.8)	73.4 (67.8–79.1)	39.93 ± 7.66	34.27 ± 4.51	43.04 ± 1.30	47.57 ± 1.31	52.23 ± 1.89	
**Perception of the protection period of COVID-19 vaccine (month)**								< 0.001
< 1	1,275 (4.3)	85.0 (83.1–87.0)	44.35 ± 6.13	36.04 ± 3.52	43.01 ± 1.38	47.54 ± 0.91	52.23 ± 1.95	
1	3,311 (11.0)	82.0 (80.7–83.3)	42.47 ± 5.87	36.40 ± 3.08	43.01 ± 1.44	47.44 ± 1.04	51.90 ± 1.80	
3	7,807 (26.1)	86.5 (85.8–87.3)	43.98 ± 6.17	36.55 ± 3.02	43.06 ± 1.41	47.49 ± 1.08	52.34 ± 1.92	
6	10,590 (35.4)	92.5 (92.0–93.0)	45.52 ± 5.94	36.70 ± 3.15	43.19 ± 1.38	47.49 ± 1.08	52.42 ± 1.95	
12	5,084 (17.0)	94.0 (93.3–94.7)	45.84 ± 5.93	36.68 ± 3.13	43.21 ± 1.39	47.54 ± 1.09	52.37 ± 1.91	
Unclear	1,858 (6.2)	87.6 (86.1–89.1)	42.60 ± 6.22	36.11 ± 3.67	43.00 ± 1.38	47.36 ± 1.06	52.26 ± 1.93	

COVID-19, Corona Virus Disease 2019; NPV, New Pneumonia Virus; Quartile of lifestyle score: Q1, scoring 12–40; Q2, scoring 41–45; Q3, scoring 46–49; Q4, scoring 50–56.

### Association between lifestyles and COVID-19 vaccination coverage rate

**Figure 3:**
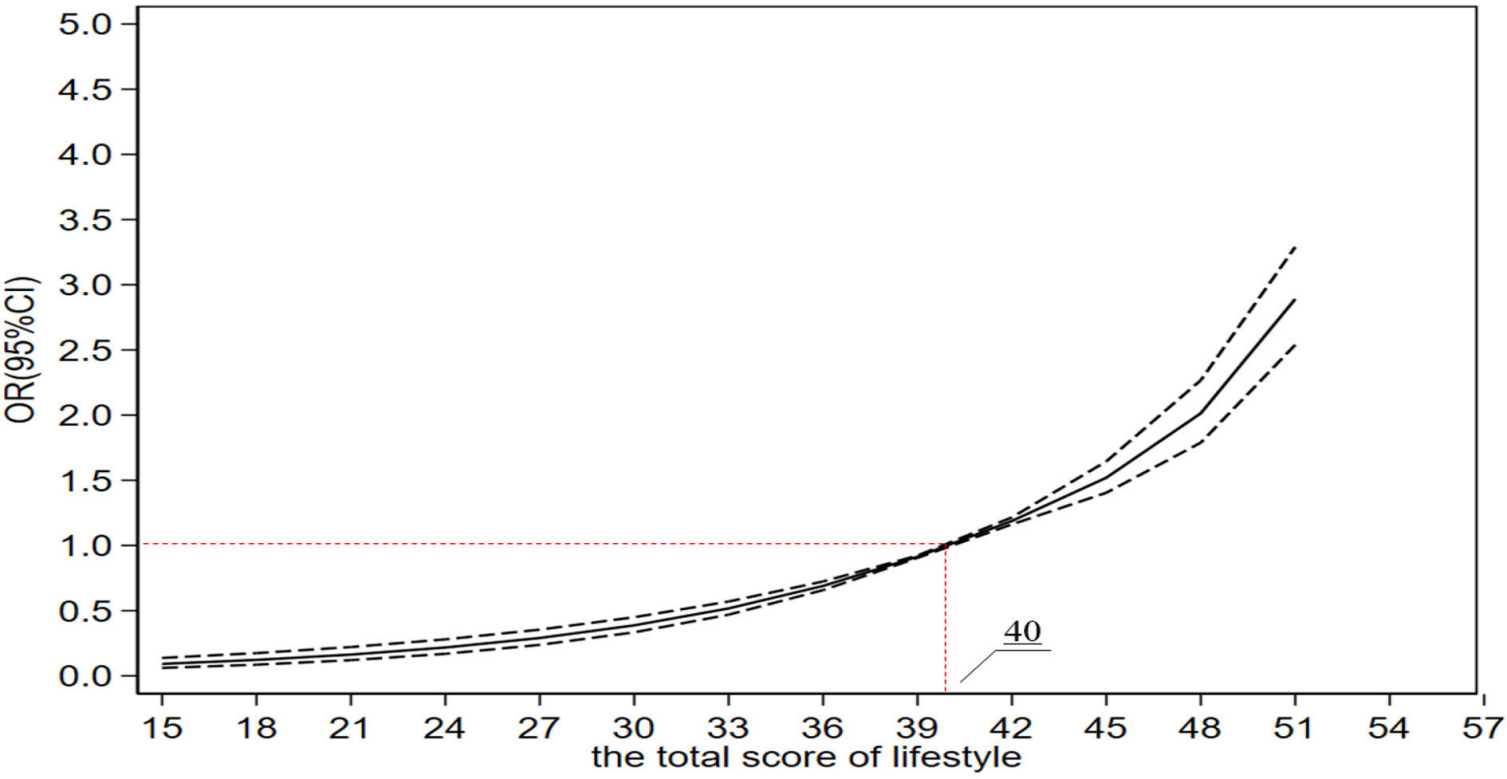
The association between lifestyle score and the COVID-19 vaccination.

After adjusting for sociodemographics and COVID-19 perceptions, COVID-19 vaccination coverage rate increased with lifestyle total scores [OR_*Q*2_ = 1.901 (1.718–2.103). *P* < 0.001; OR_*Q*3_ = 2.373 (2.099–2.684), *P* < 0.001; and OR_*Q*4_ = 3.765 (3.209–4.417), *P* < 0.001] (see Table 2 for details). As shown in Figure 3, the COVID-19 vaccination coverage rate increased with the lifestyle score. When the score exceeded 40, the rate of COVID-19 vaccination coverage increased rapidly, while when the score fell below 40, the rate of COVID-19 vaccination coverage dropped sharply. After adjusting for gender, age, marital status, occupation, education level, and COVID-19 perceptions, we found that maintaining a healthy body weight was a positive predictor of COVID-19 vaccination [odds ratio (OR) = 1.417 (1.039–1.932), *P* = 0.028] as maintaining a healthy diet [OR = 1.426 (1.116–1.823), *P* = 0.005]. It was also found that regular physical exercises [OR = 1.636 (1.281–2.088), *P* < 0.001], adequate sleep [OR = 3.090 (2.231–4.280), *P* < 0.001], frequent handwashing [OR = 2.357 (1.534–3.622), *P* < 0.001], using hand sanitizer [OR = 3.511 (2.396–5.144), *P* < 0.001], as well as mask wearing [OR = 2.694 (1.836–3.953), *P* < 0.001] were all significant predictors for COVID-19 vaccination coverage rates. In addition, social distancing [OR = 2.183 (1.639–2.907), *P* < 0.001], abstaining from gathering activities [OR = 1.513 (1.317–1.737), *P* < 0.001], never smoking [OR = 2.428 (2.201–2.680), *P* < 0.001], and never drinking [OR = 1.324 (1.196–1.465), *P* < 0.001] were all found to be a significant predictor for COVID-19 vaccination coverage rates (see Table 2 for details).

**Table 2:** The associations between lifestyle score, 12 items of lifestyles, and COVID-19 vaccination coverage rates.

**Lifestyle score (Quartile)**	**Unadjusted OR (95% CI)**	***P*-Value**	**Adjusted OR a (95% CI)**	***P*-Value**
Q1	Ref.		Ref.	
Q2	3.118 (2.841–3.421)	< 0.001	1.901 (1.718–2.103)	< 0.001
Q3	4.900 (4.384–5.477)	< 0.001	2.373 (2.099–2.684)	< 0.001
Q4	9.488 (8.204–10.973)	< 0.001	3.765 (3.209–4.417)	< 0.001
**Item 1. Healthy body weight**				
Never	Ref.		Ref.	
Little	1.132 (0.835–1.534)	0.425	0.909 (0.648–1.275)	0.582
Sometimes	0.905 (0.683–1.199)	0.488	0.876 (0.639–1.201)	0.410
Often	1.252 (0.949–1.651)	0.112	0.996 (0.730–1.360)	0.981
Always	2.517 (1.909–3.317)	< 0.001	1.417 (1.039–1.932)	0.028
**Item 2. Healthy diet**				
Never	Ref.		Ref.	
Little	0.835 (0.654–1.065)	0.146	0.930 (0.714–1.213)	0.595
Sometimes	0.706 (0.564–0.883)	0.002	0.837 (0.655–1.069)	0.154
Often	1.115 (0.891–1.395)	0.342	1.110 (0.869–1.418)	0.405
Always	2.084 (1.663–2.610)	< 0.001	1.426 (1.116–1.823)	0.005
**Item 3. Regular physical exercises**				
Never	Ref.		Ref.	
Little	1.298 (1.033–1.632)	0.025	1.078 (0.838–1.387)	0.558
Sometimes	1.275 (1.027–1.584)	0.028	1.093 (0.861–1.389)	0.465
Often	1.795 (1.444–2.231)	< 0.001	1.394 (1.095–1.774)	0.007
Always	2.807 (2.252–3.499)	< 0.001	1.636 (1.281–2.088)	< 0.001
**Item 4. Adequate sleep**				
Never	Ref.		Ref.	
Little	2.578 (1.918–3.466)	< 0.001	1.858 (1.323–2.608)	< 0.001
Sometimes	2.898 (2.191–3.835)	< 0.001	1.933 (1.400–2.669)	< 0.001
Often	4.512 (3.412–5.967)	< 0.001	2.438 (1.766–3.365)	< 0.001
Always	7.152 (5.388–9.493)	< 0.001	3.090 (2.231–4.280)	< 0.001
**Item 5. Regular physical examination**				
Never	Ref.		Ref.	
Little	1.098 (0.899–1.341)	0.360	0.930 (0.747–1.158)	0.518
Sometimes	0.933 (0.772–1.127)	0.473	0.899 (0.728–1.110)	0.321
Often	1.078 (0.892–1.303)	0.436	0.954 (0.770–1.181)	0.666
Always	1.900 (1.566–2.306)	< 0.001	1.222 (0.982–1.552)	0.072
**Item 6. Washing hands**				
Significantly decrease	Ref.		Ref.	
Decrease	0.672 (0.443–1.019)	0.061	0.747 (0.463–1.203)	0.230
No change	1.534 (1.054–2.232)	0.025	1.037 (0.673–1.596)	0.870
Increase	2.949 (2.033–4.278)	< 0.001	1.488 (0.969–2.285)	0.069
Significantly increase	7.018 (4.835–10.186)	< 0.001	2.357 (1.534–3.622)	< 0.001
**Item 7. Using sanitizers**				
Significantly decrease	Ref.		Ref.	
Decrease	0.944 (0.646–1.379)	0.765	1.082 (0.703–1.668)	0.720
No change	2.549 (1.826–3.558)	< 0.001	1.521 (1.038–2.230)	0.032
Increase	4.471 (3.212–6.226)	< 0.001	2.144 (1.465–3.136)	< 0.001
Significantly increase	10.971 (7.870–15.294)	< 0.001	3.511 (2.396–5.144)	< 0.001
**Item 8. Wearing masks**				
Significantly decrease	Ref.		Ref.	
Decrease	0.737 (0.502–1.081)	0.118	0.836 (0.544–1.286)	0.415
No change	0.992 (0.701–1.404)	0.962	0.942 (0.637–1.393)	0.765
Increase	2.618 (1.861–3.683)	< 0.001	1.604 (1.092–2.356)	0.016
Significantly increase	7.346 (5.233–10.313)	< 0.001	2.694 (1.836–3.953)	< 0.001
**Item 9. Attending gathering activities**				
Significantly increase	Ref.		Ref.	
Increase	0.328 (0.290–0.371)	< 0.001	0.598 (0.522–0.686)	< 0.001
No change	0.333 (0.295–0.376)	< 0.001	0.564 (0.492–0.646)	< 0.001
Decrease	1.247 (1.105–1.407)	< 0.001	1.213 (1.061–1.387)	0.005
Significantly decrease	1.977 (1.741–2.246)	< 0.001	1.513 (1.317–1.737)	< 0.001
**Item 10. Social distancing**				
Significantly decrease	Ref.		Ref.	
Decrease	1.314 (1.005–1.718)	0.046	1.344 (0.986–1.833)	0.061
No change	1.584 (1.238–2.027)	< 0.001	1.274 (0.957–1.697)	0.097
increase	2.579 (2.020–3.292)	< 0.001	1.633 (1.229–2.169)	0.001
Significantly increase	4.785 (3.739–6.123)	< 0.001	2.183 (1.639–2.907)	< 0.001
**Item 11. Smoking**				
Regular	Ref.		Ref.	
Quit	2.304 (1.730–2.391)	< 0.001	1.536 (1.286–1.834)	< 0.001
Never	4.169 (3.850–4.515)	< 0.001	2.428 (2.201–2.680)	< 0.001
**Item 12. Drinking**				
Regular	Ref.		Ref.	
Quit	0.908 (0.760–1.085)	0.289	0.914 (0.749–1.117)	0.380
Never	2.160 (1.975–2.364)	< 0.001	1.324 (1.196–1.465)	< 0.001

OR, odds ratio; 95% CI, 95% Confidence Interval.

Quartile of lifestyle score: Q1, scoring 12–40; Q2, scoring 41–45; Q3, scoring 46–49; Q4, scoring 50–56.

a, adjusted for gender, age, marital status, educational level, occupation, perceptions of the NPV mutation, the effectiveness of COVID-19 vaccine, and the protection period of COVID-19 vaccine.

### Propensity score matching analysis for COVID-19 vaccination and lifestyles

After PSM (see Annexure 4: Table 2), we captured a balanced sample consisting 2,041 pairs of respondents (a total of 4,082 respondents) with comparable gender, age, marital status, educational level, occupation, and perception of COVID-19. The COVID-19 vaccination rates among Chinese respondents living specific lifestyles were examined based on these homogeneous respondents after PSM. In detail, the COVID-19 vaccination rates for participants who reported healthy body weight and healthy diet were 55.1% (52.8–57.4%) and 55.5% (52.9–58.2%), respectively, while the rates for those who took regular physical exercises and those who reported adequate sleep were 55.7% (52.7–58.7%) and 57.9% (55.0–60.9%), respectively. Participants with significantly increased frequency of washing hands, using sanitizer, wearing masks, and keeping social distance had COVID-19 vaccination coverage rate of 57.7% (55.4–60.1%), 58.2% (55.8–60.6%), 55.2% (53.2–57.2%), and 54.9% (52.3–57.6%), respectively. COVID-19 vaccination coverage rates were 58.1% (55.0–61.2%) among those who abstained from gathering activities, while for those who never smoked nor consumed alcohol were 56.2% (54.1–58.4%) and 53.6% (50.8–56.5%), respectively (see Table 3 for details).

**Table 3:** The COVID-19 vaccination rates among 12 items of lifestyle pre- and post-PSM.

**Covariates**	**Pre-PSM**			***P*-Value**	**Post-PSM**			***P*-Value**
	**Total participants (proportion, %)**	**Vaccinated participants**	**Vaccination rate (%, 95% CI)**		**Total participants (proportion, %)**	**Vaccinated participants**	**Vaccination rate (%, 95% CI)**	
**Healthy body weight**				< 0.001				< 0.001
Always	15,732 (52.6)	14,629	93.0 (92.6–93.4)		1,802 (44.1)	993	55.1 (52.8–57.4)	
Often	8,167 (27.3)	7,092	86.8 (86.1–87.6)		1,277 (31.3)	600	47.0 (44.2–49.7)	
Sometimes	4,057 (13.6)	3,354	82.7 (81.5–83.8)		698 (17.1)	303	43.4 (39.7–47.1)	
Little	1,574 (5.2)	1,348	85.6 (83.9–87.4)		243 (6.0)	114	46.9 (40.6–53.2)	
Never	395 (1.3)	332	84.1 (80.4–87.7)		62 (1.5)	31	50.0 (37.2–62.8)	
**Healthy diet**				< 0.001				< 0.001
Always	12,643 (42.2)	11,834	93.6 (93.2–94.0)		1,351 (33.1)	750	55.5 (52.9–58.2)	
Often	8,385 (28.1)	7,435	88.7 (88.0–89.3)		1,202 (29.4)	601	50.0 (47.2–52.8)	
Sometimes	5,870 (19.6)	4,884	83.2 (82.2–84.2)		1,040 (25.5)	463	44.5 (41.5–47.5)	
Little	2,257 (7.5)	1,928	85.4 (84.0–86.9)		382 (9.4)	174	45.5 (40.5–50.6)	
Never	770 (2.6)	674	87.5 (85.2–89.9)		107 (2.6)	53	49.5 (39.9–59.2)	
**Regular physical exercises**				< 0.001				< 0.001
Always	9,912 (33.1)	9,233	93.1 (92.7–93.6)		1,081 (26.5)	602	55.7 (52.7–58.7)	
Often	8,579 (28.7)	7,694	89.7 (89.0–90.3)		1,192 (29.2)	623	52.3 (49.4–55.1)	
Sometimes	7,451 (24.9)	6,413	86.1 (85.3–86.9)		1,176 (28.8)	525	44.6 (41.8–47.5)	
Little	3,346 (11.2)	2,887	86.3 (85.1–87.4)		522 (12.8)	237	45.4 (41.1–49.7)	
Never	637 (2.1)	528	82.9 (80.0–85.8)		111 (2.7)	54	48.6 (39.2–58.1)	
**Adequate sleep**				< 0.001				< 0.001
Always	10,192 (34.1)	9,522	93.4 (92.9–93.9)		1,086 (26.6)	629	57.9 (55.0–60.9)	
Often	10,395 (34.7)	9,352	90.0 (89.4–90.5)		1,421 (34.8)	728	51.2 (48.6–53.8)	
Sometimes	7,023 (23.5)	5,984	85.2 (84.4–86.0)		1,159 (28.4)	508	43.8 (41.0–46.7)	
Little	2,082 (6.9)	1,742	83.7 (82.1–85.3)		370 (9.1)	164	44.3 (39.2–49.4)	
Never	233 (0.8)	155	66.5 (60.4–72.6)		46 (1.1)	12	26.1 (12.9–39.3)	
**Regular physical examination**				< 0.001				< 0.001
Always	9,439 (31.5)	8,779	93.0 (92.5–93.5)		1,005 (24.6)	537	53.4 (50.3–56.5)	
Often	8,008 (26.8)	7,071	88.3 (87.6–89.0)		1,177 (28.8)	595	50.6 (47.7–53.4)	
Sometimes	7,094 (23.7)	6,152	86.7 (85.9–87.5)		1,120 (27.4)	536	47.9 (44.9–50.8)	
Little	4,256 (14.3)	3,766	88.5 (87.5–89.4)		627 (15.4)	298	47.5 (43.6–51.4)	
Never	1,128 (3.7)	987	87.5 (85.6–89.4)		153 (3.8)	75	49.0 (41.0–57.0)	
**Washing hands**				< 0.001				< 0.001
Significantly increase	16,577 (55.4)	15,607	94.1 (93.8–94.5)		1,689 (41.4)	975	57.7 (55.4–60.1)	
Increase	9,050 (30.2)	7,884	87.1 (86.4–87.8)		1,506 (36.9)	711	47.2 (44.7–49.7)	
No change	3,749 (12.5)	2,919	77.9 (76.5–79.2)		755 (18.4)	309	40.9 (37.4–44.4)	
Decrease	414 (1.4)	251	60.6 (55.9–65.4)		109 (2.8)	37	33.9 (24.9–43.0)	
Significantly decrease	135 (0.5)	94	69.6 (61.8–77.5)		23 (0.5)	9	39.1 (17.6–60.7)	
**Using sanitizer**				< 0.001				< 0.001
Significantly increase	15,844 (52.9)	14,955	94.4 (94.0–94.7)		1,639 (40.2)	954	58.2 (55.8–60.6)	
Increase	9,184 (30.7)	8,015	87.3 (86.6–88.0)		1,482 (36.3)	705	47.6 (45.0–50.1)	
No change	4,329 (14.5)	3,447	79.6 (78.4–80.8)		834 (20.4)	340	40.8 (37.4–44.1)	
Decrease	416 (1.4)	246	59.1 (54.4–63.9)		101 (2.5)	33	32.7 (23.4–42.0)	
Significantly decrease	152 (0.5)	92	60.5 (52.7–68.4)		26 (0.6)	9	34.6 (15.0–54.2)	
**Wearing masks**				< 0.001				< 0.001
Significantly increase	21,059 (70.4)	19,753	93.8 (93.5–94.1)		2,301 (56.4)	1,270	55.2 (53.2–57.2)	
Increase	6,281 (21.0)	5,298	84.3 (83.5–85.2)		1,134 (27.8)	533	47.0 (44.1–49.9)	
No change	1,971 (6.6)	1,323	67.1 (65.0–69.2)		515 (12.6)	200	38.8 (34.6–43.1)	
Decrease	458 (1.5)	276	60.3 (55.8–64.8)		101 (2.5)	27	26.7 (18.0–35.5)	
Significantly decrease	156 (0.5)	105	67.3 (59.9–74.8)		31 (0.7)	11	35.5 (17.6–53.3)	
**Attending gathering activities**				< 0.001				< 0.001
Significantly increase	5,405 (18.1)	4,888	90.4 (89.7–91.2)		733 (18.0)	388	52.9 (49.3–56.6)	
Increase	2,946 (9.8)	2,228	75.6 (74.1–77.2)		673 (16.5)	279	41.5 (37.7–45.2)	
No change	3,200 (10.7)	2,428	75.9 (74.4–77.4)		679 (16.6)	255	37.6 (33.9–41.2)	
Decrease	8,389 (28.0)	7,733	92.2 (91.6–92.8)		1,021 (25.0)	552	54.1 (51.0–57.1)	
Significantly decrease	9,985 (33.4)	9,478	94.9 (94.5–95.4)		976 (23.9)	567	58.1 (55.0–61.2)	
**Social distancing**				< 0.001				< 0.001
Significantly increase	12,734 (42.6)	11,940	93.8 (93.3–94.2)		1,349 (33.1)	741	54.9 (52.3–57.6)	
Increase	9,907 (33.1)	8,819	89.0 (88.4–89.6)		1,426 (34.9)	715	50.1 (47.5–52.7)	
No change	5,404 (18.1)	4,500	83.3 (82.3–84.3)		957 (23.4)	425	44.4 (41.3–47.6)	
Decrease	1,503 (4.9)	1,210	80.5 (78.5–82.5)		277 (6.8)	126	45.5 (39.6–51.4)	
Significantly decrease	377 (1.3)	286	75.9 (71.5–80.2)		73 (1.8)	34	46.6 (34.9–58.3)	
**Smoking**				< 0.001				< 0.001
Never	18,559 (62.0)	17,510	94.3 (94.0–94.7)		1,977 (48.4)	1,112	56.2 (54.1–58.4)	
Quit	1,664 (5.6)	1,482	89.1 (87.6–90.6)		196 (4.8)	104	53.1 (46.0–60.1)	
Regular smoker	9,702 (32.4)	7,763	80.0 (79.2–80.8)		1,909 (46.8)	825	43.2 (41.0–45.4)	
**Drinking**				< 0.001				< 0.001
Never	10,361 (34.6)	9,703	93.6 (93.2–94.1)		1,182 (28.9)	634	53.6 (50.8–56.5)	
Quit	1,080 (3.6)	930	86.1 (84.0–88.2)		154 (3.8)	75	48.7 (40.7–56.7)	
Regular drinker	18,484 (61.8)	16,122	87.2 (86.7–87.7)		2,746 (67.3)	1,332	48.5 (46.6–50.4)	

95% CI, 95% Confidence interval; PSM, Propensity score matching.

## Discussion

This is a large-scale national study investigated the present lifestyle and COVID-19 vaccine coverage rate in 31 provinces across mainland China. During the COVID-19 pandemic, the majority of Chinese inhabitants maintained a healthy lifestyle (the participants scored 44,606.13), implying favorable prospects for developing herd immunity and halting the spread of COVID-19. Meanwhile, the current study discovered that 89.4% of the Chinese inhabitants have had a COVID-19 vaccination. This significant advancement in COVID-19 vaccination coverage was attributed to China's tremendous efforts, which included the following: China enacted a vaccine management law and passed the WHO assessment of its National Vaccine Management System (NRS), which ensured vaccine quality and supply (25). In addition, China and other countries exchanged technical information and collaborated on COVID-19 vaccines (26), resulting in an increase in vaccine safety and effectiveness.

Gender inequities in COVID-19 vaccination coverage rates were observed in this study. Females had a higher COVID-19 vaccination coverage rate than males, which was associated with the healthier lifestyle for females. Gender disparities have been recognized as a significant explanatory parameter for lifestyle variations (27), and females live healthier lifestyles due to their rising educational level, incomes, self-reliance, and the global ongoing promotion of healthy lifestyles (28, 29). Usually, lower vaccine coverage rate is associated with concerns on adverse reactions and side effects. Previous studies indicated that adverse reactions might occur throughout the course of COVID-19 vaccination, including pain at the injection site, fever, fatigue, headache, muscle pain, diarrhea, nausea, appetite disturbance, swelling, and cough (30). Similarly, some other studies stated that increased concerns about vaccine side effects were associated with lower rates of vaccination against COVID-19 (31). In terms of COVID-19 vaccination, females represented better physical fitness, which, in turn, enhanced adaptability to the adverse effects of vaccination, reduced concerns about COVID-19 vaccine adverse reactions and side effects, and strengthened COVID-19 vaccination practices.

Lifestyle was found to be positively associated with COVID-19 vaccination. The study discovered that healthy lifestyle was the positive factor of COVID-19 vaccination. Peoples with healthier lifestyles had higher COVID-19 vaccination rate. The positive correlation originated from that healthy lifestyle not only helps eliminate concerns about adverse reactions to COVID-19 vaccination and side effects of COVID-19 vaccines, but also contributes more contact with healthcare service providers (regular physical examination). However, it was noteworthy that contrary to the findings of this study, some previous studies suggested that better health condition was associated with lower willingness to get vaccinated (32). The reason for this paradox was that healthy lifestyles were not the same as good health condition, although they were highly correlated.

Lifestyles were important influencing factors of COVID-19 vaccination. To be specific, the study found that regardless of sociodemographics, healthy body weight as well as adequate sleep was protective factors of COVID-19 vaccination. We also found that maintaining healthy body weight and adequate sleep were positively correlated with enhanced COVID-19 vaccination behavior. After controlling for 8 covariates [age, gender, marital status, education level, occupation, perception of the NPV mutation, perception of the effectiveness of COVID-19 vaccine, and perception of the protection period of COVID-19 vaccine (month)] through PSM, groups with always maintaining healthy body weight and adequate sleep had the highest COVID-19 vaccination rates (55.1 and 57.9%, respectively). Previous studies had shown that being obese was a significant factor for the likelihood of an adverse immune response induced by the COVID-19 vaccine (33, 34), and sleep also played a vital role in health, with studies suggesting that insomnia reduced the risk of the effectiveness of immunity (35), thereby affecting the immune efficacy of the COVID-19 vaccine. In addition, washing hands, using sanitizer, wearing masks, and social distancing were similar determinants, and the study found that washing hands, using sanitizer, wearing masks, and social distancing were also positively associated with enhanced COVID-19 vaccination behavior. It was found that the COVID-19 vaccination rates of groups with these four lifestyles were also the highest after PSM (57.7, 58.2, 55.2, and 54.9%, respectively). Meanwhile, being non-smoker and not drinking alcohol were found to be determinants, and it found that compared to regular smoking and regular alcohol drinking, never smoking and drinking were positively correlated with enhanced COVID-19 vaccination behavior. After PSM, groups who never smoke or drink alcohol reported COVID-19 vaccination rates of 56.2 and 53.6%, respectively. Respondents who lived these specific lifestyles had the highest COVID-19 vaccination rates, further verifying the reliability of the findings. These influencing factors are indeed positive factors for the COVID-19 vaccination.

### Strengths and weaknesses of the study

This is the first large-scale study to draw a panorama of the current lifestyle and the prevalence of COVID-19 vaccination in a large, saturated sample of the Chinese population. Due to the saturation of the sample, we can be certain that our estimate of vaccination coverage rate is accurate and robust. To avoid the confounding factors, and explore the association between specific lifestyles and COVID-19 vaccination, we adopted the most widely accepted PSM method to capture a homogeneous sample of 2,041 pairs of participants (*n* = 4,082) from 29,925 valid respondents. One of the major limitations of the current study is that we were unable to develop a uniform framework for lifestyle assessment due to the lack of a universal scale to assess lifestyle globally COVID-19 vaccine hesitancy in China. Due to the fact that a comprehensive assessment of lifestyle during the COVID-19 pandemic can serve as an important basis for public health policy decision, the development of a global scale for lifestyle assessment will become one of the important directions of future research. The second disadvantage is that the limited number of confounding factors included in the binary logistic regression analysis. The analysis of the results of the binary logistic regression model should obviously be based on effective adjustment of confounding factors, and the number of confounding factors is definitely greater than the number of confounding factors included in this study. Another study's shortcomings include its limited number of covariates in the PSM procedure. The inference of associations between specific lifestyles and COVID-19 vaccination is based on comprehensive control for confounders, but obviously, the confounding variable set is larger than the range of confounding variables enrolled into the PSM statistical process in this article. Finally, despite the fact that we used data from a large saturation sample of the population from 31 provinces, due to the epidemic, we were forced to collect data *via* online questionnaires utilizing the snowball sampling approach, which may cause selection bias in the sample. Therefore, these research findings may differ from those estimated using probability sampling. In addition, due to the COVID-19 epidemic, this study was forced to conduct an online survey, and the influence of lifestyle on COVID-19 vaccination observed may not be applicable to persons without Internet access.

## Conclusion

In summary, the substantial proportions of the mainland Chinese are living healthy lifestyles during the COVID-19 pandemic and their COVID-19 vaccination rate is high. It is crucial to continually strengthen public awareness and education about healthy male lifestyles, as well as to improve people's knowledge about COVID-19 vaccines. It is critical to maintain healthy lifestyles, including always maintaining a healthy body weight, healthy diet, regular physical exercises, adequate sleep, regular physical examination, increasing frequency significantly in washing hands, using sanitizers, wearing masks, social distancing, decreasing social gathering activities, and to abstain from smoking and drinking, all of which will aid in reaching the timeline for herd immunity.

## Data availability statement

The raw data supporting the conclusions of this article will be made available by the authors, without undue reservation.

## Ethics statement

The studies involving human participants were reviewed and approved by the Life Science Ethics Review Committee of Zhengzhou University (Record No: 2021-01-12-05).

## Author contributions

JG and WD: conceptualization. WZ, WW, and CM: data curation. JW, YM, and BY: formal analysis. YM and MW: funding acquisition. JW, CM, and WZ: investigation. YM and YL: methodology. WD: project administration. JW, MW, and WW: resources. YM and WZ: software and writing—original draft. WD, BY, DX, and CT: writing—reviewing and editing. All authors contributed to the article and approved the submitted version.

## Funding

This study was funded by the National Social Science Fund of China (Grant No. 21BGL222) and the collaborative Innovation Key Project of Zhengzhou (Grant No. 20XTZX05015).

## Conflict of interest

The authors declare that the research was conducted in the absence of any commercial or financial relationships that could be construed as a potential conflict of interest.

## Publisher's note

All claims expressed in this article are solely those of the authors and do not necessarily represent those of their affiliated organizations, or those of the publisher, the editors and the reviewers. Any product that may be evaluated in this article, or claim that may be made by its manufacturer, is not guaranteed or endorsed by the publisher.
