# Supplementary material for: Association between lifestyle and COVID-19 vaccination: A national cross-sectional study
Source: Front Public Health. 2022 Oct 11;10:918743. doi: 10.3389/fpubh.2022.918743 (PMC9593211; doi:10.3389/fpubh.2022.918743)
Supplement: Supplementary file 1 [file Table_1.pdf]

Annexure 3: Data collection and explanatory variables

Table 1. Questionnaire about online survey questions list

**Section 1: General Information**
1. The name ______
2. Which country are you from? The origin of your ID? ______ , ______
3. What's your gender?<br>A. Male B. Female
4. How old are you? (Age) _______
5.How tall are you in centimeters? ____ cm (centimeters); What is your weight in kilograms? _____kg (kg) (Fill in the blank)
6. What's your marital status?<br>A. Unmarried B. Married C. Divorced D. Widowed E. Others
7. What's your education level?<br>A. Illiteracy B. Primary school C. Middle school D. High school E. College or above
8. What's your occupation type?<br>A. Worker B. Farmer C. Business staff D. Student E. Technical staff F. Government staff G. Retired H. No fixed occupation I. Others
9. At present, do you think the New Pneumonia Virus has mutated?<br>A. Yes B. No C. Unclear
10. Do you think vaccination against COVID-19 is effective?<br>A. Very effective B. Effective C. Not sure D. Ineffective E. Completely ineffective F. Unclear
11. How long do you think the protection of period of COVID-19 vaccine is?<br>A. <1 B. 1- C. 3- D. 6- E. 12- F. Unclear
**Section 2: Lifestyle Behaviors**
1. Do you maintain a healthy body weight?<br>A. Always B. Often C Sometimes D. Little E. Never
2. Do you maintain a healthy diet (whole food, plant-predominant diet)?

A. Always B. Often C Sometimes D. Little E. Never
3. Do you maintain a regular physical exercises (150 minutes every week)?<br>A. Always B. Often C Sometimes D. Little E. Never
4. Do you maintain an adequate sleep (≥8 hours every day)?<br>A. Always B. Often C Sometimes D. Little E. Never
5. Do you maintain a regular physical examination (4 times every year, excluding those for illness)?<br>A. Always B. Often C Sometimes D. Little E. Never
6. How does the frequency in washing hands (A normal frequency of washing hands is >10 times every day)?<br>A. Significantly increase B. Increase C. No change D. Decrease E. Significantly decrease
7. How does the frequency in using sanitizers(A normal frequency of using sanitizers is >5 times every day)?<br>A. Significantly increase B. Increase C. No change D. Decrease E. Significantly decrease
8. How does the frequency in wearing masks (A normal frequency of wearing masks is changing a mask every day)?<br>A. Significantly increase B. Increase C. No change D. Decrease E. Significantly decrease
9. How does the frequency in attending gathering activities?<br>A. Significantly decrease B. Decrease C. No change D. Increase E. Significantly increase
10. How does the frequency in social distancing (The normal social distancing is ≥1 meters)?<br>A. Significantly increase B. Increase C. No change D. Decrease E. Significantly decrease
11. Do you smoke?<br>A. Never B. Quit C. Regular
12. Do you drink?<br>A. Never B. Quit C. Regular
**Section 3: COVID-19 Vaccination**
1. Do you have received the primary COVID-19 vaccine?<br>A. Vaccinated B. Being vaccinated C. No, but preparing to receive the COVID-19 vaccine D. No, and not sure to get the COVID-19 vaccine E. No, and hesitant to receive the COVID-19 vaccine
