# Supplementary material for: Association between lifestyle and COVID-19 vaccination: A national cross-sectional study
Source: Front Public Health. 2022 Oct 11;10:918743. doi: 10.3389/fpubh.2022.918743 (PMC9593211; doi:10.3389/fpubh.2022.918743)
Supplement: Supplementary file 2 [file Table_2.pdf]

Annexure 4: PSM procedure

Table 2. The Sample balances before PSM and after PSM

**Covariates**	**Unmatched / Matched**	**χ2**	**P value**
Gender	U	125.934	<0.001
	M	0.016	0.900
Age	U	91.324	<0.001
	M	8.517	0.074
Marital status	U	919.249	<0.001
	M	9.471	>0.05
Educational level	U	987.778	<0.001
	M	4.301	0.367
Occupation	U	326.655	<0.001
	M	7.743	0.459
Perception of the NPV mutation	U	848.642	<0.001
	M	1.449	0.485
Perception of the effectiveness of COVID-19 vaccine	U	2621.518	<0.001
	M	0.714	0.982
Perception of the protection period of COVID-19 vaccine	U	510.325	<0.001
	M	2.761	0.737

PSM procedures:

1)grouping: The unvaccinated participant group was set as “control group”, n=3172 (10.6%); the vaccinated participant group was set as “intervention group”, n=26753(89.4%)

2) matching method: 1:1 matching.

3) confounders: Gender, Age, Marital status, Educational level, Occupation, Perception of the NPV mutation, Perception of the effectiveness of COVID-19 vaccine, Perception of the protection period of COVID-19 vaccine.

4) outcome: 2041 pairs of participants captured (n=4082).
