# Supplementary figures and images for: Association between lifestyle and COVID-19 vaccination: A national cross-sectional study
Source: Front Public Health. 2022 Oct 11;10:918743. doi: 10.3389/fpubh.2022.918743 (PMC9593211; doi:10.3389/fpubh.2022.918743)

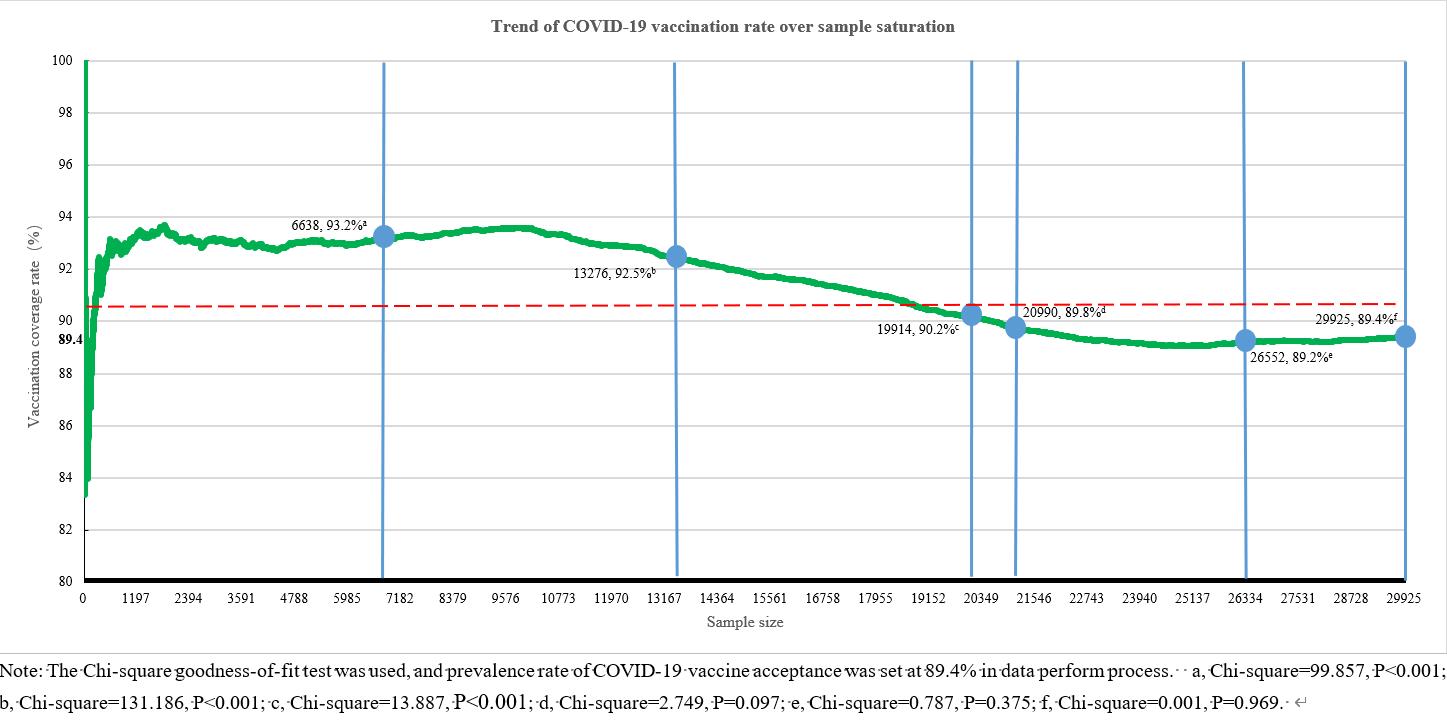

Supplement: Supplementary file 3 [file Image_1.jpg]

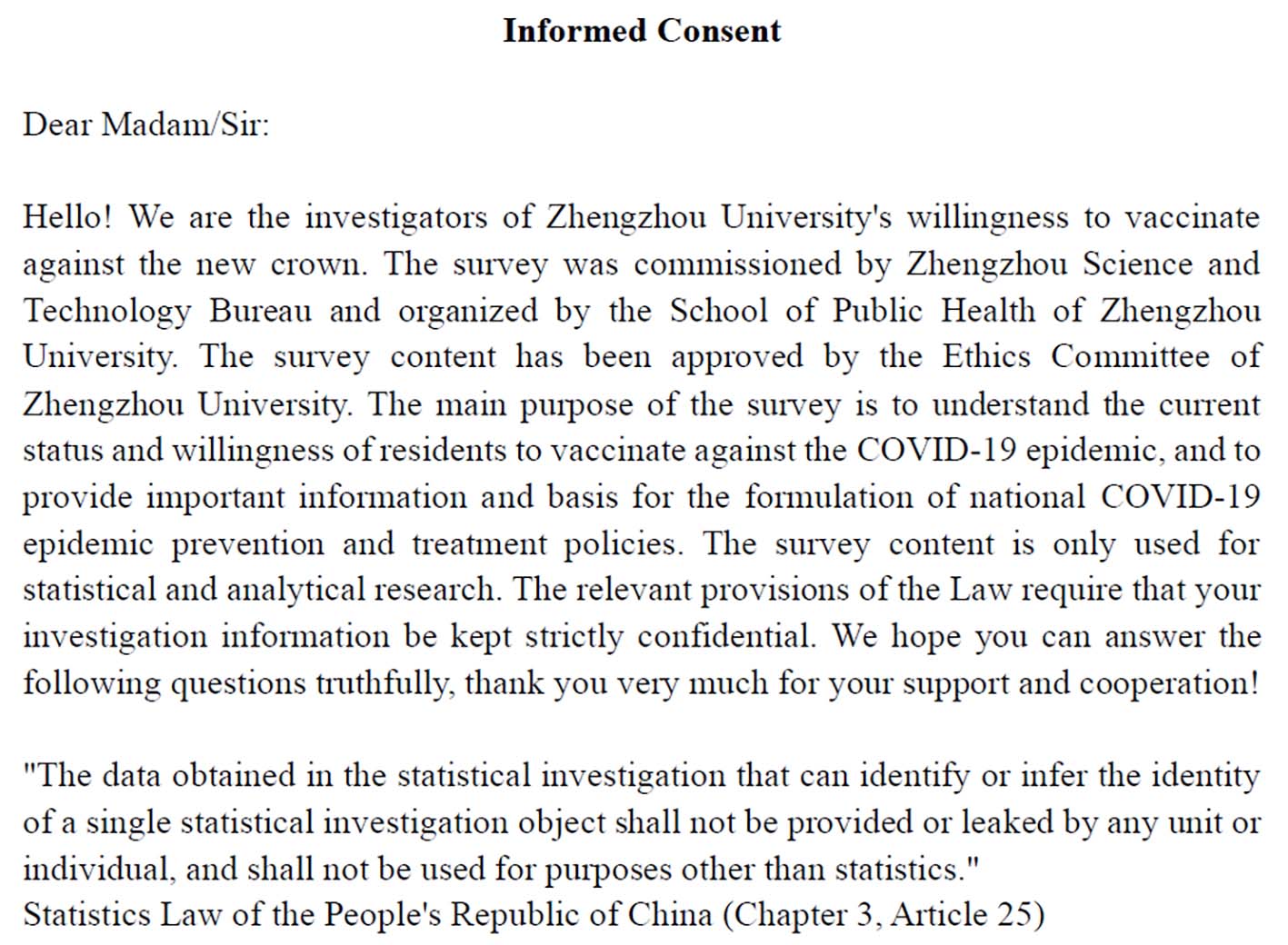

Supplement: Supplementary file 4 [file Image_2.jpg]
